# Mitochondrial Fragmentation Due to Inhibition of Fusion Increases Cyclin B through Mitochondrial Superoxide Radicals

**DOI:** 10.1371/journal.pone.0126829

**Published:** 2015-05-22

**Authors:** Tejas M. Gupte

**Affiliations:** 1 National Centre for Biological Sciences (NCBS-TIFR), UAS-GKVK campus, Bellary road, Bangalore, 560 065, Karnataka, India; 2 inStem, Institute for Stem Cell Biology and Regenerative Medicine, GKVK post, Bellary road, Bangalore, 560 065, Karnataka, India; Children's Hospital of Pittsburgh, University of Pittsburgh Medical Center, UNITED STATES

## Abstract

During the cell cycle, mitochondria undergo regulated changes in morphology. Two particularly interesting events are first, mitochondrial hyperfusion during the G_1_-S transition and second, fragmentation during entry into mitosis. The mitochondria remain fragmented between late G_2_- and mitotic exit. This mitotic mitochondrial fragmentation constitutes a checkpoint in some cell types, of which little is known. We bypass the ‘mitotic mitochondrial fragmentation’ checkpoint by inducing fragmented mitochondrial morphology and then measure the effect on cell cycle progression. Using *Drosophila* larval hemocytes, *Drosophila* S2R^+^ cell and cells in the pouch region of wing imaginal disc of *Drosophila* larvae we show that inhibiting mitochondrial fusion, thereby increasing fragmentation, causes cellular hyperproliferation and an increase in mitotic index. However, mitochondrial fragmentation due to over-expression of the mitochondrial fission machinery does not cause these changes. Our experiments suggest that the inhibition of mitochondrial fusion increases superoxide radical content and leads to the upregulation of cyclin B that culminates in the observed changes in the cell cycle. We provide evidence for the importance of mitochondrial superoxide in this process. Our results provide an insight into the need for mitofusin-degradation during mitosis and also help in understanding the mechanism by which mitofusins may function as tumor suppressors.

## Introduction

Mitochondrial morphology changes in concert with the cell cycle, and steady-state morphology is maintained by fission and fusion [1]. Mitochondria are tubular in G_1_-, consisting of filamentous structures disconnected from each other [2]. At the G_1_-S transition, all the isolated elements of the mitochondrial reticulum form a hyperfused giant network that is electrically connected [3]. The formation of this mitochondrial network correlates with a transient increase in the amount of cyclin E, which in turn advances the cell cycle from G_1_- into S-phase. In late S-phase, the hyperfused mitochondrial network fragments into tubules [2,3]. In late G_2_-, the mitochondria are seen as thick filaments. At the G_2_/M transition, prior to nuclear envelope breakdown, the mitochondria undergo fission into small fragments [2,3]. This mitotic fragmentation is mediated by specific, post-translational modification of key proteins involved in mitochondrial fission as well as mitochondrial fusion. Dynamin-related protein Drp1 is a GTPase that executes mitochondrial fission [4]. At the G_2_/M transition, a SUMO protease SenP5 translocates from the nucleoli to the mitochondria where it deSUMOylates Drp1 promoting the formation of pro-fission oligomers [5]. The fission activity of Drp1 is increased by phosphorylation of Ser-585 by the mitotic cyclin complex containing cyclin B and Cdk1 [2]. Along with an increase in fission, mitochondrial fusion is inhibited. Various proteins have been isolated that mediate fusion of the mitochondrial outer membrane and separately of the mitochondrial inner membrane. Among these, mitofusin (Mfn) proteins are of particular interest because they contain a GTPase domain, a coiled-coil domain for tethering their counter-parts on opposing mitochondria as well as a bi-partite transmembrane domain anchoring them to the mitochondrial outer membrane [6]. Mammalian cells possess two mitofusins, Mfn1 and Mfn2, of which Mfn1 is specific to the mitochondria. MARCH5 is an E3 ubiquitin ligase. During G_2_/M MARCH5-mediated ubiquitylation of Mfn1 increases, consequently Mfn1 levels are reduced [7]. Increase in pro-fission activity of Drp1, and the loss of the pro-fusion protein Mfn1, result in mitotic mitochondrial fragmentation.

Drp1-mediated fragmentation of the mitochondrial network is an essential step in apoptosis that is conserved across phyla [8]. However, the significance of fragmented mitochondrial morphology during mitosis is not completely understood. Inhibition of mitotic mitochondrial fragmentation has cell-type specific phenotypes [3,9,10], suggesting that at least in some cells mitotic mitochondrial fragmentation could constitute a cell-cycle checkpoint. The operational details of this proposed checkpoint are obscure. Lack of mitochondrial fission causes replicative stress activating the G_2_/M checkpoint by ATM kinase [9] or caspase-8 dependent apoptosis at the G_2_/M checkpoint [10].

A similar compartment-based G_2_/M checkpoint is the Golgi mitotic checkpoint that has been characterized to a greater extent. Golgi ribbon severing is brought about by the activity of BARS [11] and GRASP65 [12]. Blocking the activity of BARS (using dominant-negative or antibody) or of GRASP65 (siRNA) leads to reduced recruitment and impaired activation of Aurora-A at the centrosome [13], which in turn prevents activation of cyclin B-Cdk1 and hence functions as a checkpoint. This G_2_/M checkpoint is bypassed by the over-expression of Aurora-A [13]. Using a similar thought-process, we have modulated the mitochondrial fission-fusion machinery to bypass the mitochondrial morphology component of the G_2_/M checkpoint.

We find that mitochondrial fragmentation brought about by inhibition of fusion is important in mitosis. Prolonged inhibition of fusion, as performed in our assays by RNA interference, increases the mitotic index by upregulating cyclin B in a mitochondrial superoxide-dependent manner. We discuss the implications of this in normal cell cycle control as well as in cancer.

## Materials and Methods

*Drosophila* stocks, chemicals and antibodies are listed (S1, S2, S3 Tables). Primer pairs with a binding site for T7 RNA polymerase were used to amplify 500–1000 bp of *gfp* or *marf*. Double-stranded RNA (dsRNA) was synthesized using the MEGAscript T7 Kit (Ambion). Concentrations of dsRNA were estimated by absorbance, aliquoted and stored at -20°C.

*Drosophila* strains were grown in cornmeal agar at 25°C. Overexpression and dsRNA-mediated depletion of transgenes (mentioned in S1 Table) was achieved in hemocytes by crossing the UAS-transgene bearing virgin females to males containing the hemocyte-specific *collagen Gal4* [14]. Larval hemocytes were derived from the progeny of these crosses as described [15,16] and stained to visualize mitochondria [15]. Overexpression in the wing disc was accomplished by using males containing *scalloped Gal4* [17] as a transgene. S2R^+^ cells were cultured in Schneider’s *Drosophila* medium supplemented with 7.5% heat-inactivated fetal bovine serum, benzylpenicillin, streptomycin and L-glutamine. S2R^+^ cells were treated with 1 μg dsRNA in 12-well cell culture dishes (Greiner bio-one) at 10^6^/well over 5 days and plated in clean, uncoated coverslip dishes for microscopy. For flow cytometry, cells were harvested from three 25 cm^2^ tissue culture flasks (Nunc # 136196), which were originally seeded with 3 x 10^6^ cells per flask. Upon adherence, 3 μg dsRNA in 3 ml serum-free medium (Express Five SFM) was added for 45 min and replaced with 3 μg dsRNA in 3 ml growth medium for 72 h. Cells were allowed a 20-h recovery in growth medium without dsRNA and the dsRNA treatment was repeated. Doubling time of cells was measured by the online tool (Roth V. 2006 http://www.doubling-time.com/compute.php).

### Microscopy

Cells were plated on Goldseal coverglass No. 1, cleaned and sterilized by UV treatment. These cells were imaged using a 100x, 1.4 NA objective on Nikon TE2000-U inverted microscope equipped with Cascade 512B EM-CCD camera (Roper Scientific) controlled by Metamorph software (Molecular Devices). Excitation and emission wavelengths were achieved by using the appropriate filters from Chroma Technology Corp (Bellows Falls, Vermont, USA). Pixel size for acquisition of images was 90 nm. Images were processed using Metamorph or ImageJ (WCIF or FIJI) and represented using Adobe Photoshop. For S2R^+^ cells, well separated, non-blebbing, healthy looking cells with one nucleus and without large inclusions, as selected from white light transmission were imaged. These constituted 70–80% of the cell population. Mitochondrial morphology in S2R^+^ cells was imaged by staining the cells with 20nM MitoTracker DeepRed in growth medium for 15 minutes. To prevent selection bias for mitochondrial morphology, all fluorescence images were captured and cells that satisfied the criteria above were quantified. Mitochondrial morphology was scored as tubular or fragmented, depending on whether most of the mitochondrial area was occupied by tubular mitochondria. Cells with almost equal proportions were categorized as intermediate. Well-separated, dome-shaped hemocytes with no visible yeast (that can come from dissecting the *Drosophila* larva) were selected for imaging and quantification. Mitochondrial morphology in hemocytes was visualized by mito-GFP fluorescence [18] and categorized as described earlier [15] or by 2 nM MitoTracker DeepRed in complete medium for 15 minutes. For nuclear content correlation with mitochondrial morphology, hemocytes were incubated in Hoechst 33342 for 15 minutes, and the mitochondria were marked by genetically encoded mitoGFP. Single-exposure images were acquired in white light transmission, UV exposure (350 nm ± 25 nm excitation, 435 nm long-pass emission) and GFP exposure (480 nm ± 20 nm exposure, 535 nm ± 25 nm emission). The mitoGFP image was used to interpret mitochondrial morphology. DNA content was calculated from the integrated intensity of the nuclear fluorescence in the Hoechst image using ImageJ (Rasband, W.S., ImageJ, U. S. National Institutes of Health, Bethesda, Maryland, USA, http://imagej.nih.gov/ij/, 1997–2014). Across the larval genotypes within an experiment, exposure for the Hoechst (DNA content) was constant, and hence these intensities correlated with relative DNA content.

For detection of superoxide, hemocytes were stained with 2 μM dihydroethidium in growth medium for 15 min, following precautions and protocol as described [19]. Unbound probe was washed away with excess medium. Single-exposure fluorescence images were acquired as mentioned above, using excitation wavelength of 530 nm ± 15 nm and emission wavelength 575 nm ± 20 nm. The same field was then imaged using white light transmission to determine cell boundaries. The fluorescence image was background-corrected by subtracting the intensity of non-cellular regions in the same image from each pixel using ImageJ. Total fluorescence intensity of each hemocyte was computed in ImageJ by drawing outline of the cell on the transmission image, and transposing the region to the background-subtracted image of dihydroethidium fluorescence and calculating the integrated intensity.

Circulating hemocyte counts and phospho (Ser10) Histone H3 staining of hemocytes were performed as described [14,20]. Mitotic index is defined as the number of phosphohistone H3-positive cells per 100 cells counted., and atleast 500 cells were counted. Immunohistochemistry of wing discs was performed as described [21]. Anti-phospho (Ser10) Histone H3 (Millipore #06–570) was used at a dilution of 1:500 for immunofluorescence of hemocytes and wing disc.

### Flow cytometry

Flow cytometry was performed using on a BD Calibur system. Cell cycle analysis was performed with minor modifications from the protocol described for S2 cells [22]. For analysis of cell-cycle distributions, data from 30,000 single cell events were plotted as frequency histograms and automated cell cycle analysis was performed using FlowJo software (Tree Star). For cyclin B and phosphohistone H3 analysis, cells were fixed (1% paraformaldehyde or 95% chilled ethanol respectively) and permeabilized (0.1% Tween-20 or 0.25% Triton-X-100 respectively), incubated with antibody (anti- cyclin B, DSHB # F2F4 supernatant—1:10 and anti-phospho (Ser10) Histone H3, Millipore #06–570–1:100) and stained with propidium iodide. Cyclin B and phosphohistone H3 were detected by secondary antibodies conjugated with AlexaFluor 488. Controls were stained with secondary antibody alone. At least 10,000 single-cell events were acquired for analysis.

BrdU pulse-chase was performed with minor modifications from published protocols [23,24]. Following 3 days of dsRNA mediated depletion S2R^+^ cells were resuspended, and plated in fresh culture flasks with exposure to an additional round of dsRNA to prevent the induction of quiescence [23]. 48 hours after this, cells were resuspended and harvested at 500xg. These cells were incubated with 60 μM BrdU in the dark, at 25°C for 1 hour. Cells were either harvested immediately (0 hours chase) or they were harvested, and grown in medium without BrdU for defined chase period. For BrdU detection, cells were harvested at 1000xg, washed with 500 μl ice-cold 1x HBSS. The cells were fixed and permeabilized using an ice-cold mixture of 1 ml 95% ethanol (in water) and 100 μl 1x HBSS. For subsequent steps, cells were pelleted at 3000xg. The ethanol-treated cells were rehydrated in 1x HBSS for 5 minutes at ambient temperature, pelleted, treated with 2 N HCl in blocking buffer (1x HBSS containing 0.1% Tween-20 and 1% BSA) for 30 minutes at 25°C. The acid-hydrolyzed cells were pelleted and washed with blocking solution. Finally the cell-pellet was incubated with 1:10 dilution of anti-BrdU supernatant (DSHB #G3G4) in blocking buffer for 30 minutes at 25°C in the dark. After washing the cells in blocking buffer twice, they were incubated with 1:250 dilution of donkey-anti mouse IgG tagged with Alexa488 for 20 minutes at 25°C in the dark. The cells were washed with blocking buffer and 1x HBSS. Finally they were resuspended in 1 ml solution containing 875 μl 1x HBSS, 100 μl propidium iodide staining solution (0.5 mg/ml in 5 mM trisodium citrate) and 25 μl of 1 mg/ml heat-treated RNaseA, and used for flow cytometry. At least 10,000 single cell events were acquired for analysis. Controls were cells not exposed to BrdU but treated with all other reagents identically, including anti-BrdU primary and Alexa488 conjugated secondary antibody. BrdU, analysis were performed using FlowJo software (Tree Star). Relative movement [RM] and transit time through S-phase cells were calculated and plotted as previously described [25–27].

## Results

### *Drosophila* hemocytes recapitulate changes in mitochondrial morphology associated with cell cycle phases

**Fig 1:**
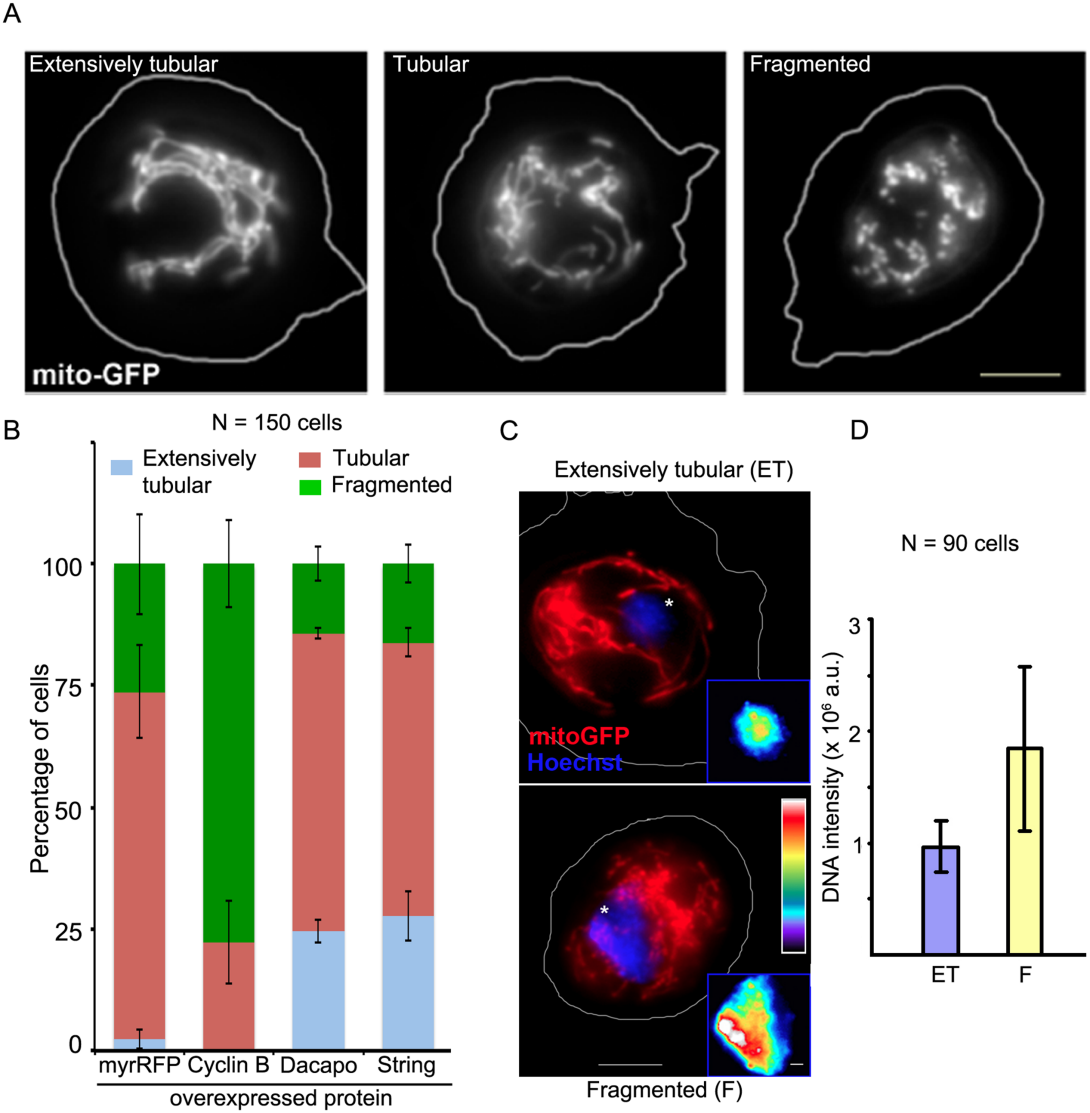
*Drosophila* larval hemocytes display cell cycle correlated changes in mitochondrial morphology. Mitochondria in hemocytes from third instar *Drosophila* larvae are visualized using mito-GFP under the control of collagenGAL4. (A) The hemocytes are classified on the basis of mitochondrial morphology as ‘extensively tubular’, ‘tubular’ or ‘fragmented’. (B) Overexpression of cell cycle regulators changes the distribution of mitochondrial morphology. In control hemocytes, expressing mito-GFP and myr-RFP under control of *collagenGAL4*, 2.3 ± 2.0% cells have extensively tubular mitochondrial morphology, 71.5 ± 9.6% are classified as displaying tubular mitochondrial morphology and 26.2 ± 10.3% as fragmented. Overexpression of cyclin B eliminates the extensively tubular population; cells displaying tubular mitochondrial morphology are reduced to 22.3 ± 8.5% while cells displaying fragmented mitochondrial morphology increase to 77.6 ± 8.9%. The overexpression of Dacapo shows the following distributions 24.5 ± 2.3%—extensively tubular, 61.1 ± 1% tubular and 14.3 ± 3.6% fragmented. The overexpression of String shows the following distributions 27.7 ± 5.2%—extensively tubular, 56.2 ± 3% tubular and 16.1 ± 3.8% fragmented. The overexpression of Dacapo and String increases the extensively tubular population. The histogram shows distribution of mitochondrial morphology (mean ± SEM) from three independent experiments with at least 50 cells per experiment. Differences are significant by t-test (control versus cyclin B, *p<0*.*05*, control versus Dacapo, *p<0*.*01*, control versus String, *p<0*.*05*). (C) Hemocytes derived from cyclin B overexpressing larvae have fragmented mitochondria (recorded by MitoTracker DeepRed) and twice the DNA content (measured by fluorescence of Hoechst 33342), lower image, compared to hemocytes from Dacapo overexpressing larvae that display extensively tubular mitochondria, upper image. The differences in nuclear fluorescence can be compared from equally scaled, pseudo-olored images (insets), where warmer colors indicate greater DNA fluorescence. (D) Hoechst 33342 fluorescence (quantified using ImageJ) doubles from 0.96 x 10^6^ ± 0.22 x 10^6^ arbitrary units (a.u.) in extensively tubular cells to 1.84 x 10^6^ ± 0.73 x 10^6^ a.u. in the fragmented cells. Scale bars A, C, 5 μm, and C inset 1 μm.

Cell cycle-associated changes in mitochondrial morphology have been characterized in mammalian cells. Highly tubular mitochondria are observed during the G_1_-S transition and fragmented mitochondria during mitotic stages. [2,3,5,28]. Regulators of the cell cycle have been characterized in *Drosophila* [29–33] (summarized in S1 Fig). We found that modulation of these cell cycle regulators in *Drosophila* in larval hemocytes affects mitochondrial morphology in a predictable manner. On the basis of mitochondrial morphology, we categorized *Drosophila* hemocytes (Fig 1A) as extensively tubular, tubular or fragmented [15]. Most of the hemocytes from control larvae had tubular mitochondrial morphology; a few had fragmented mitochondrial morphology while only a minority displayed extensively tubular mitochondrial morphology (Fig 1B). Ectopic over-expression of cyclin B, the mitotic cyclin, caused most of the hemocytes to display fragmented mitochondrial morphology (Fig 1B). On the other hand, ectopic over-expression of the p27 ortholog Dacapo [33] or of the Cdc25-type phosphatase String, increased greatly the fraction of cells with extensively tubular mitochondrial morphology (Fig 1B). To correlate the mitochondrial morphology observed in *Drosophila* hemocytes with the cell cycle phase they were in, we measured DNA content from the fluorescence of Hoechst 33342 and recorded the mitochondrial morphology in the same cell, with ectopic over-expression of either cyclin B or Dacapo. Cyclin B over-expressing hemocytes that had fragmented mitochondria (Fig 1C) contained twice the amount of nuclear DNA (Fig 1D) compared to Dacapo-over-expressing hemocytes possessing extensively tubular mitochondria (Fig 1C and 1D). These data suggest that ectopic expression of cell cycle regulators in *Drosophila* hemocytes influences the cell cycle and hence the mitochondrial morphology. Together, these experiments show that *Drosophila* larval hemocytes recapitulate the extensively tubular morphology of the G_1_-S transition and mitotic fragmentation associated with cell cycle progression. Hence *Drosophila* hemocytes will be used as a model system to determine how mitochondrial fragmentation affects the cell cycle.

### Inhibiting mitochondrial fusion increases mitotic index

**Fig 2:**
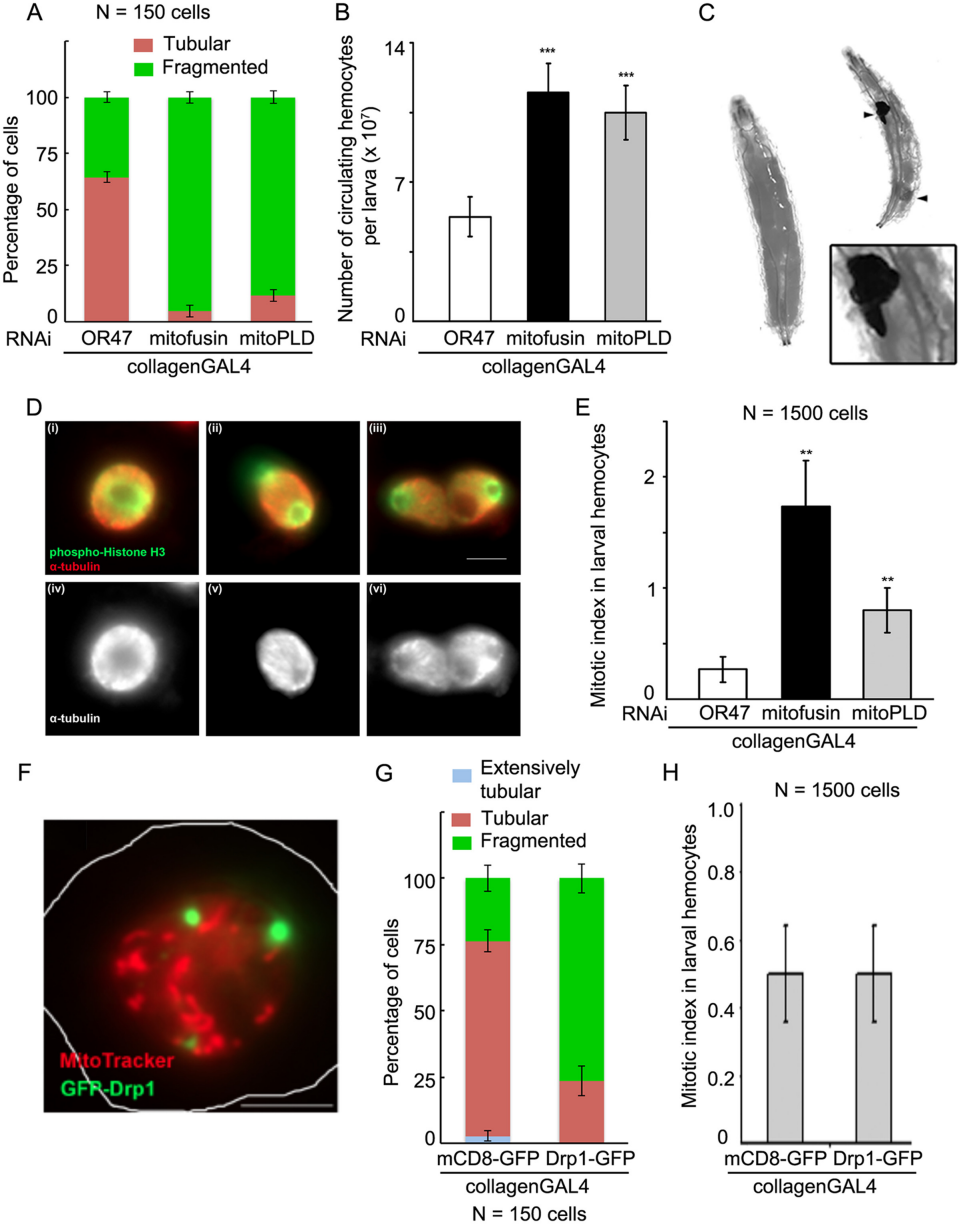
Depletion of mitochondrial fusion proteins increases cell number and mitotic index. (A) *Drosophila* homolog of mitofusin or of mitoPLD was depleted by expressing the UAS-RNAi construct in larval hemocytes using *collagenGAL4*, mitochondrial morphology was imaged by MitoTracker DeepRed staining and classified (as illustrated in Fig 2). In the controls, i.e., cells depleted of the unrelated gene OR47 using *collagenGAL4*, 64.4 ± 2.3% cells have tubular mitochondrial morphology and 35.6 ± 2.3% cells have fragmented mitochondrial morphology. Upon depleting mitofusin 95.3 ± 2.7% cells, and on depleting mitoPLD 88.2 ± 2.7% cells display fragmented mitochondrial morphology. (B) The number of circulating hemocytes per larva was estimated using a Neubauer chamber. Control larvae contain 5300 ± 900 circulating hemocytes per larva. Depletion of mitofusin (*collagenGAL4>marf RNAi*) increases this number to 11480 ± 1480, while depletion of mitoPLD (*collagenGAL4>mitoPLD RNAi*) increases it to 10490 ± 1360 circulating hemocytes per larva. Histogram represents data from 3 independent experiments with 2 to 6 larvae per genotype per experiment. The differences are significant by t-test, *p<0*.*01*. (C) Third instar larvae excessively depleted of mitofusin (*collagenGAL4/collagenGAL4>marf RNAi/ marf RNAi*, right) displayed melanotic masses (arrowhead) consistent with an increase in cell number, while control larvae (CS-Bz, left) did not show such masses. Inset shows a magnified view of one of these melanotic masses. (D) Mitotic hemocytes were detected by phospho (Serine-10) histone H3 labeling, and the stage within mitosis detected by relative arrangement of the tubulin spindle and the chromatin- (i, iv) metaphase, (ii, v) anaphase, (iii, vi) telophase. (E) Correlating with the number of circulating hemocytes, controls have low mitotic index (0.27 ± 0.13). Depletion of mitofusin in the hemocytes increased the mitotic index by 5-fold to 1.73 ± 0.41, while depletion of mitoPLD increased it by 3-fold to 0.8 ± 0.19. The increases are significant (*p<0*.*05*, *t*-test). (F, G) *Drosophila* larval hemocytes overexpressing GFP-Drp1 (green) have fragmented mitochondria (MitoTracker DeepRed, red). (H) *Drosophila* larval hemocytes possessing fragmented mitochondria due to Drp1 overexpression have the same mitotic index as cells from control larvae (overexpressing mCD8-GFP) and containing tubular mitochondria. Thus, fragmenting mitochondria by increased fission does not increase mitotic index.

Mitochondrial morphology is maintained by a balance of fission and fusion [1], allowing mitochondrial fragmentation to be achieved either by inhibiting fusion or by increasing fission. We inhibited mitochondrial fusion by depleting the *Drosophila* homologs of either mitofusin (Marf) [34] or of mitochondrial phospholipase D (mitoPLD) [35,36] using *GAL4* and *UAS-RNAi* to achieve cell-type specific depletion of the gene product by RNA interference. The expression of the double-stranded construct in larval hemocytes was achieved by using *collagenGAL4* that has been previously used to ectopically express gene-products in these cells [14]. Depletion of mitofusin or of mitoPLD produced mitochondrial fragmentation (Fig 2A). To test the effect of this morphological change on the cell cycle, we estimated the number of circulating hemocytes in these larvae. Depletion of mitofusin or mitoPLD doubled the number of circulating larval hemocytes compared to control larvae (Fig 2B). This increase in cell number was consistent with an increase in mitosis. Excessive depletion of mitofusin using two copies of both the *GAL4* and the *RNAi* construct produced melanotic masses in larvae (Fig 2C), similar to those observed upon over-expression of Ras [14]. To verify the mitotic origin of the phenotype (increase in cell number), we labeled hemocytes for the mitotic marker phospho-(Serine-10) Histone H3 (hereafter phosphohistone H3, Fig 2D) and obtained the mitotic index. The mitotic index increased 6-fold upon depletion of mitofusin and 3-fold upon depletion of mitoPLD (Fig 2E).

Next we probed if mitochondrial fragmentation achieved by increasing fission also produced the same phenotype. Mitochondria were fragmented in hemocytes over-expressing GFP-Drp1 (Fig 2F and 2G) but this did not affect the mitotic index (Fig 2H). Thus increased mitochondrial fragmentation by itself does not seem to affect the cell cycle.

**Fig 3:**
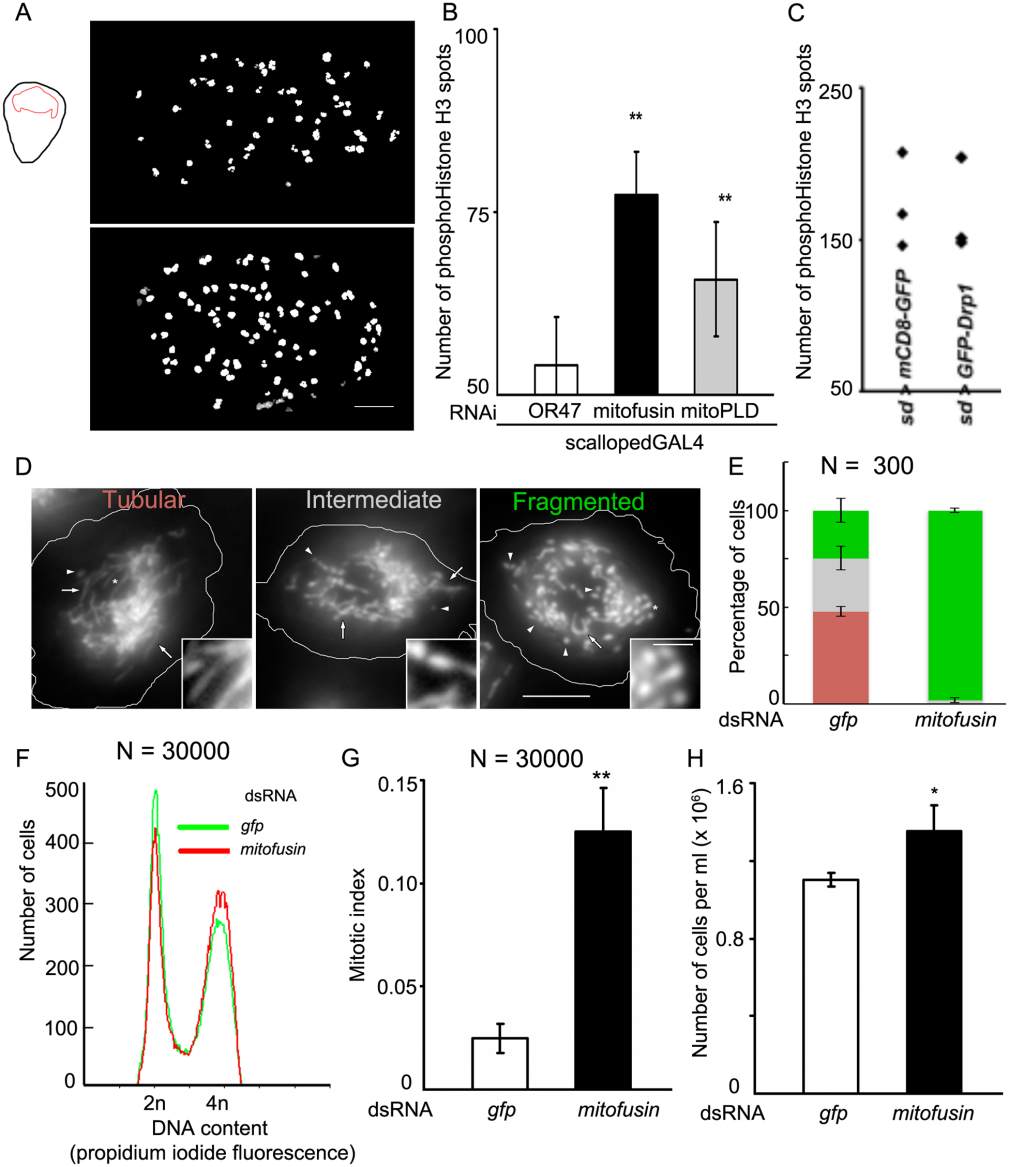
Mitochondrial fusogens regulate cell cycle across cell types. (A) Punctae of phosphoHistone H3 counted in the proliferating wing pouch region of *Drosophila* third instar larval wing disc. Mitofusin depleted wing pouch (*scallopedGAL4*>*mitofusin* RNAi) contains more phosphoHistoneH3 punctae than control wing pouch (*scallopedGAL4*>*OR47* RNAi, top). (B) Control wing pouches contain 54 ± 7 punctae. Depletion of mitofusin increases the number of punctae to 77 ± 6 and the depletion of mitoPLD increases this to 65 ± 7. Values are mean ± SEM from 3 larave per genotype and the increase in number is significant (*p<0*.*05*, Mann-Whitney test). (C) Overexpression of GFP-Drp1 in the wing pouch (*scallopedGAL4*>*GFP-Drp1*) does not change the number of phosphoHistone H3-positive spots compared to controls overexpressing mCD8-GFP (*scallopedGAL4*>*mCD8-GFP*). The overexpression of GFP itself can increase the number of punctae. (D) S2R^+^ cells are classified as tubular or fragmented, depending on the predominant kind of mitochondria. Cells with almost equal proportions of fragments and tubules are classified as intermediate. (E) S2R^+^ cells treated with mock dsRNA (targeting GFP) are predominantly tubular (48.77 ± 4.83%), a minority display fragmented mitochondria (19.31 ± 4.71%), while the remaining are classified as intermediate (31.93 ± 1.9%). S2R^+^ cells treated with dsRNA targeting mitofusin are fragmented (98.1 ± 1.2% fragmented). Numbers are mean ± SEM from three experiments with at least 100 cells per treatment per experiment, color scheme as in D. Fragmentation is significant (*p<0*.*01*, t-test). (F) The depletion of mitofusin increases the proportion of cells in the G_2_/M phase of cell cycle by 8%, compared to controls (gfp dsRNA treated cells). (G) The depletion of mitofusin increases the mitotic index of these cells from 0.025 ± 0.007 (gfp dsRNA treated controls) to 0.125 ± 0.021 and (H) also increases the cell number from 1.1 x 10^6^ ± 0.035 x 10^6^ to 1.35 x 10^6^ ± 0.13 x 10^6^. Differences are significant (F, *p*<0.05, Mann-Whitney test, G, H, *p<0*.*05*, *t*-test).

The robustness of these observations was tested further in the wing pouch region of the wing imaginal disc of *Drosophila* larvae. Mitofusin or mitoPLD was depleted in the wing pouch using *scallopedGAL4* [17]. This led to a 1.5-fold increase in mitotic phosphohistone H3 punctae (Fig 3A and 3B). As in the larval hemocytes, over-expression of GFP-Drp1 did not change the number of phosphohistone H3 punctae as compared to GFP over-expression controls (Fig 3C).

Since larval hemocytes could not be maintained as long-term cultures, mechanistic investigations were performed in the *Drosophila* S2R^+^ cell line. In S2R^+^ cells, mitochondrial morphology was characterized as tubular, intermediate or fragmented (Fig 3D and 3E) with most cells in the G_1_- and G_2_/M populations (Fig 3F) as analyzed by DNA content. Depletion of mitofusin by introduction of dsRNA into the cells caused mitochondrial fragmentation (Fig 3E) and increased the percentage of cells in G_2_/M by ~7% (Fig 3F), increased the mitotic index by 6-fold (Fig 3G) and also increased the cell density (Fig 3H). From a starting density of 1 x 10^5^ cells/ml, control cells grew to 1.1 x 10^6^ cells/ml with a doubling time of ~ 35 h. Cells depleted of mitofusin had a small but significant reduction in the doubling time to ~ 32 h, reaching a density of 1.35 x 10^6^ cells/ml in the same time.

These data show that across cell and tissue types, mitochondrial fragmentation by inhibiting mitochondrial fusion increases mitotic index and cell number. However, mitochondrial fragmentation achieved by increasing fission does not affect cell cycle.

### Mitofusin depletion perturbs cell cycle progression

**Fig 4:**
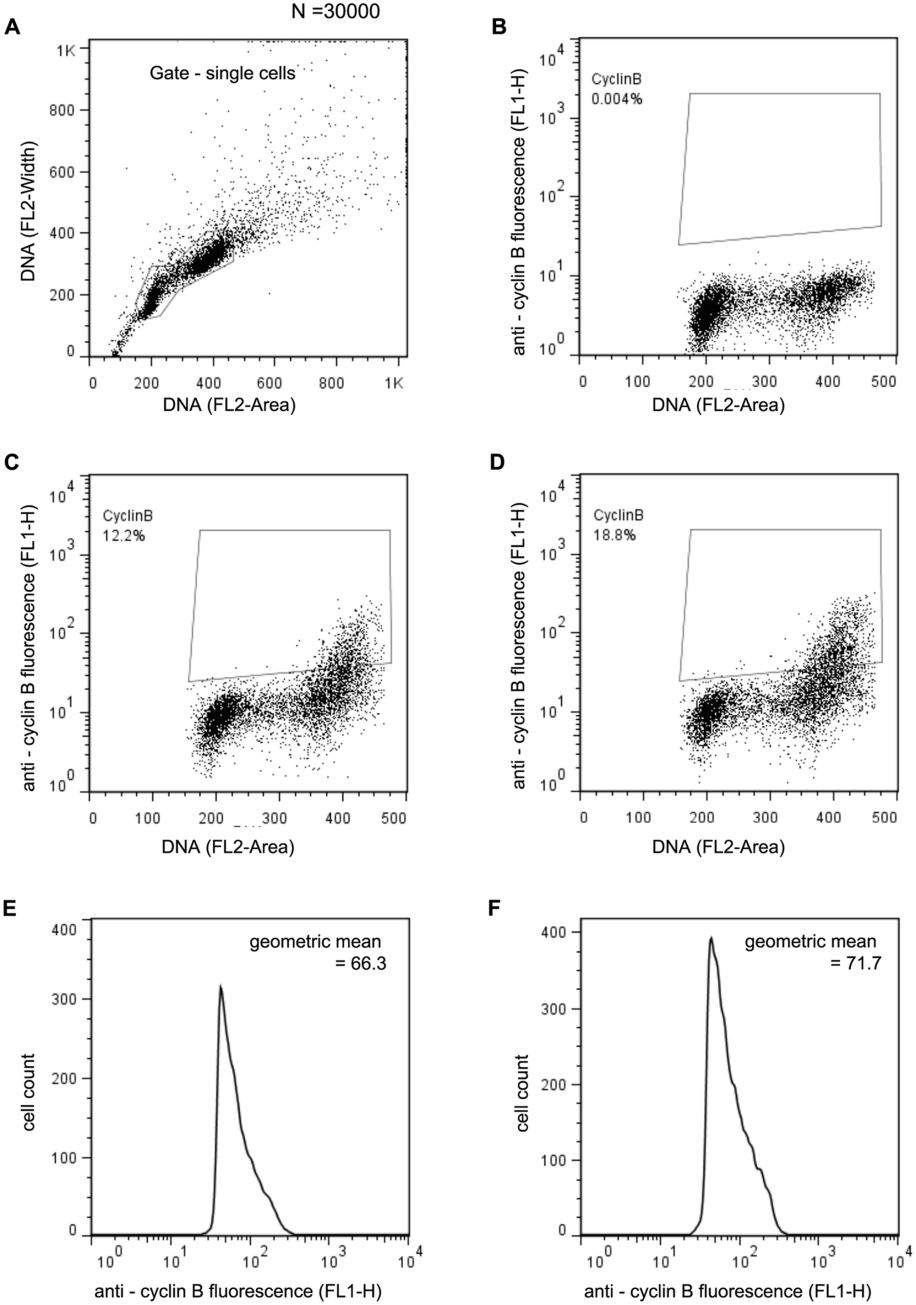
Inhibition of mitochondrial fusion influences mitosis through cyclin B. (A) Doublet-discrimination module operating on all events to identify single cells with unreplicated and replicated DNA on the basis of pulse area and pulse width using the DNA stained by propidium iodide to generate a signal. Gate identifies single cells used for further analysis. (B) Negative control for the cyclin B staining, shows S2R^+^ cells treated with secondary antibody but not the primary anti-cyclin B. Fluorescence detected (Y-axis) is non-specificty, and is used to determine the gate for the cyclin B positive population. (C) Controls (S2R^+^ cells treated with gfp dsRNA) contain 12.2% cyclin B positive cells. (D) In S2R^+^ cells depleted of mitofusin, the population of cyclin B positive cells increases to 18.8%. (E, F) The content of cyclin B in the positive population (gates in C, D) increases from 66.3 a.u (controls, E) to 71.7 a.u. upon depletion of mitofusin (F).

We observed a mitotic phenotype, upon depletion of mitofusin. Cyclin B is responsible for progression of the cell cycle through the G_2_/M checkpoint into mitosis. Hence we tested for the involvement of cyclin B in this context (Fig 4). In S2R^+^ cells, depletion of mitofusin caused ~7% increase in the cyclin B-positive population (Fig 4C and 4D). Within this population, the level of cyclin B (geometric mean of anti-cyclin B fluorescence in the cyclin B positive population) also increased (Fig 4E and 4F).

**Fig 5:**
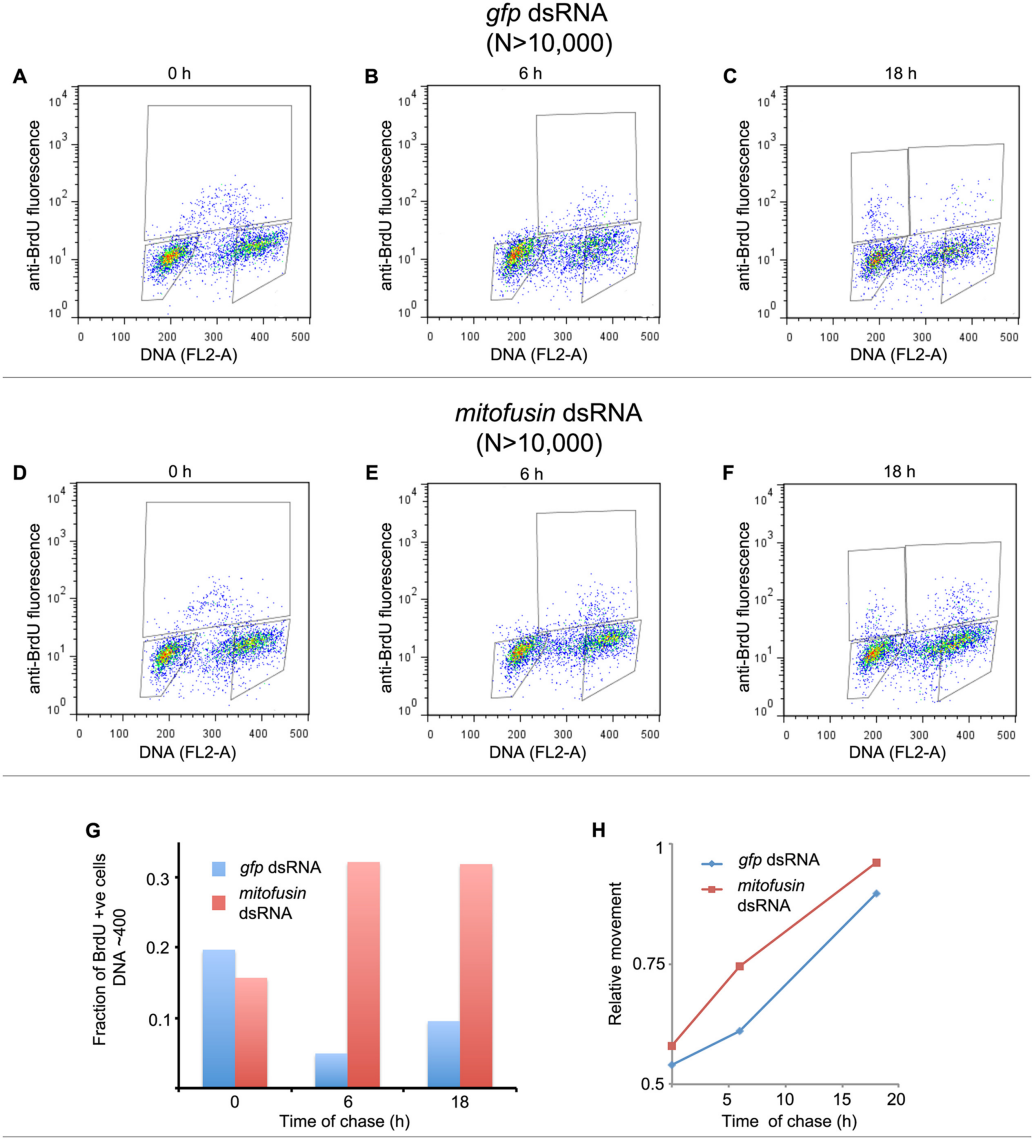
Inhibiting mitochondrial fusion affects cell cycle progression. (A-F) Representative histograms of asynchronous cultures of S2R^+^ cells treated with gfp dsRNA (A-C) or mitofusin dsRNA (D-F), pulse-labeled with BrdU and harvested (A, D 0h chase) or pulsed with BrdU and cultured in BrdU-free medium for 6 h chase (B, E) or for 18 h chase (C, F). Cells were harvested at the indicated times and analyzed by flow cytometry to determine kinetics of transit through different phases of the cell cycle. Boxes are labeled to indicate the cell cycle phase they represent. (G) In control cells (blue) the BrdU labeled population transits through mitosis while the mitofusin depleted cells (red) and delayed in exiting mitosis. (H) Relative movement of BrdU positive undivided cells through S-phase is faster in mitofusin depletion as compared to controls, accounting for the net decrease in cell cycle duration. Values are data from a representative experiment with 10,000 cells per sample.

To understand how the change in cyclin B content affected the cell cycle and cell number, we performed BrdU pulse-chase analysis (Fig 5). S-phase cells were labeled by the hour-long pulse (Fig 5A and 5D) and the BrdU positive *gfp* dsRNA treated cells show a gradual cell cycle progression (Fig 5A, 5B and 5C). The BrdU-labeled mitofusin-depleted cells accumulated in mitosis (Fig 5D, 5E, 5F, and 5G). Interestingly, measurement of relative movement [RM] [25,26] indicated that the mitofusin-depleted cells had a greater value of RM than the *gfp* dsRNA-treated control cells (Fig 5H). Hence the mitofusin-depleted cells transited faster through the S-phase (Table 1) and also had a shorter doubling time (32 h as compared to 35 h for controls, as calculated from cell densities earlier).

**Table 1:** Kinetics of cell cycle progression.

	Relative movement (RM)		Time for S-phase transit (*t*_*S*_) (h)		Doubling time (h)
	*Duration of chase*		*Duration of chase*		
**Treatment**	*6 h*	*18 h*	*6 h*	*18 h*	
Control (gfp dsRNA)	0.61 ± 0.01	0.90 ± 0.03	27.2	22.8	34.6
mitofusin dsRNA	0.75 ± 0.07	0.96 ± 0.01	12.2	19.6	31.9

### Mitochondrial superoxide concentration influences cell number

**Fig 6:**
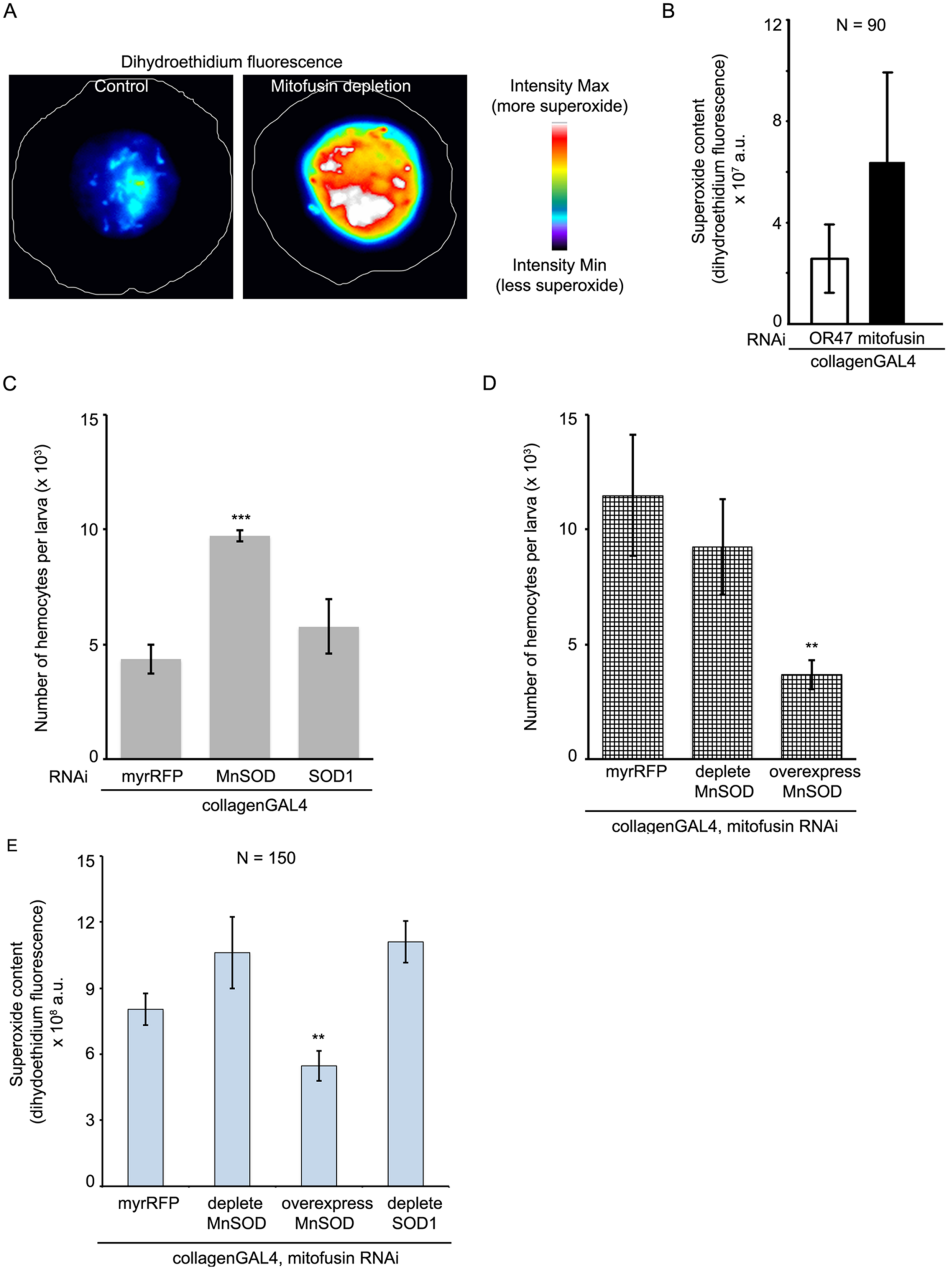
Mitochondrial superoxide governs cell number. (A) Pseudo-colored images of *Drosophila* larval hemocytes derived from control (*collagenGAL4>OR47 RNAi*) and mitofusin depletion (*collagenGAL4>marf RNAi*) and stained with dihydoethidium to monitor superoxide content. Warmer colors in the images corresponding to higher intensities in the LUT bar match greater dihydroethidium fluorescence indicating greater superoxide. (B) Depletion of mitofusin increases the total fluorescence per cell from 2.5 x 10^7^ ± 1.3 x 10^7^ a.u. to 6.3 x 10^7^ ± 3.5 x 10^7^ a.u. Values are mean ± SD from a single experiment with 30 cells per genotype. (C) Control larvae contain 4350 ± 647 circulating hemocytes. Depleting the mitochondrial MnSOD increases this number to 9716 ± 240 circulating hemocytes per larva, however depleting cytosolic SOD (SOD1) does not change the circulating hemocyte number significantly (5766 ± 1186). Values are mean ± SEM from 3 experiments, containing 2–5 larvae per genotype per experiment. Increase in cell number for MnSOD RNAi is significant (*p<0*.*05*, t-test). (D) Mitofusin depleted larvae, also expressing RFP, contain 11488 ± 2630 circulating hemocytes. Depletion of MnSOD along with depletion of mitofusin does not increase the cell numbers further (9240 ± 2100 circulating hemocytes per larva). However, the overexpression of MnSOD in larval hemocytes depleted of mitofusin restores the cell number (3675 ± 650) to wild-type levels. Values are mean ± SEM from 3 experiments, containing 2–5 larvae per genotype per experiment. Decrease in cell number for MnSOD overexpression is significant (*p<0*.*05*, t-test). (E) Depletion of mitofusin and overexpression of MnSOD decreases the total superoxide content (as inferred from dihydroethidium fluorescence), from 8 x 10^8^ a.u. ± 1.5 x 10^8^ a.u. (the depletion of mitofusin alone) to 5.4 x 10^8^ ± 0.7 x 10^8^. Values are mean ± SD from a three experiments with 50 cells per genotype.

Since mitochondrial fragmentation induced by Drp1 over-expression does not increase the mitotic index (Figs 2 and 3) it means that mitochondrial morphology by itself does not influence mitotic progression. We hypothesized that instead, a metabolic output (ATP, ROS or Ca^2+^) of mitochondrial function influences the cell cycle. Consistent with this hypothesis, inhibition of fusion is known to result in dysfunctional mitochondria and an accumulation of reactive oxygen species (ROS) [37,38]. Upon staining for several ROS species, we found that superoxide levels, as detected by dihydroethidium fluorescence, were greatly increased upon depletion of mitofusin (Fig 6A and 6B). To determine if increased superoxide could affect the cell cycle, we depleted the two superoxide dismutase (SOD) enzymes in *Drosophila* larval hemocytes using *collagenGAL4* and *UAS-RNAi* [39]. SOD converts superoxide to hydrogen peroxide. SOD1 is localized to the cytosol while MnSOD is localized to the mitochondria. Depletion of the MnSOD led to an increase in circulating hemocyte number, but depletion of SOD1 did not affect the cell number (Fig 6C). Thus, increasing mitochondrial superoxide generation causes an increase in cell number. It is therefore likely that increase in superoxide content by inhibiting fusion may be the primary reason for the cell cycle defects observed. We confirmed this by modulating MnSOD protein in the background of mitofusin depletion in hemocytes (Fig 6D). In this experiment, a single copy of the *collagenGAL4* was driving expression from two separate *UAS* constructs. Hence, the controls also had a similar genotype, where *collagenGAL4* caused depletion of mitofusin as well as the over-expression of myr-RFP. These larvae had increased circulating hemocytes. Over-expression of MnSOD in cells depleted of mitofusin led to a reduction in the number of circulating hemocytes (Fig 6D). Measurement of superoxide revealed that hemocytes depleted of mitofusin and overexpressing MnSOD had significantly lesser superoxide than depletion of mitofusin or of mitofusin and either superoxide dismutase as a combination (Fig 6E). Taken together, these results support the idea that mitochondrial superoxide is the signal responsible for the cell cycle changes described above, and mitochondrial fragmentation without an increase in superoxide is not sufficient to drive G_2_/M transition.

## Discussions

We initiated a study to test if fragmentation of mitochondrial morphology constituted a component of the G_2_/M checkpoint, in a manner similar to Golgi fragmentation [11–13]. Using known regulators of the cell cycle, and correlating the mitochondrial morphology with DNA content, we show that *Drosophila* larval hemocytes are a tractable system to investigate the cell cycle. Cyclin B degradation is required for mitotic exit and the over-expression of cyclin B leads to an increase in anaphase cells and delay in exiting mitosis [32]. When we over-expressed cyclin B, the hemocytes accumulated in mitosis, explaining the duplicated DNA and the fragmented mitochondria. Over-expression of Dacapo prevents entry into S-phase in the *Drosophila* larval wing disc [40]. Hemocytes over-expressing Dacapo accumulated at the G_1_-S transition, hence the extensively tubular mitochondria. Conversely, overexpression of String accelerates the G_2_/M transition but lengthens G_1_- and delays the G_1_-S transition [31,32] producing cells with G_1_-S like mitochondrial morphology.

The depletion of mitochondrial fusogenic proteins (mitofusin or mitoPLD) led to mitochondrial fragmentation and an increase in mitotic index and cell number. While the overexpression of the fission protein Drp1 caused mitochondrial fragmentation, it did not replicate the cell cycle phenotype caused by depletion of the fusogenic proteins. This indicates that at least in *Drosophila* cells, change in mitochondria from tubular morphology to fragmented morphology does not constitute a G_2_/M checkpoint.

However the inhibition of mitochondrial fusion can affect the cell cycle. We find multiple changes in the cell cycle upon depletion of mitofusin. These are—increased cyclin B content, delayed mitotic exit, and shortening of the duration of the S-phase. Together, these result in a greater number of cells being detected as mitotic events, and a reduced total cell cycle time (T_C_). The increase in hemocyte number observed upon depletion of mitofusin could be reproduced by down-regulation of MnSOD, but not by down-regulation of SOD1. This implicates increased levels of superoxide as the driving factor for the cell cycle perturbation. This interpretation is further strengthened by the observation that increased cell number due to mitofusin depletion is rescued by overexpression of MnSOD in this background.

Mfn2 and Opa1 are proteins required for the fusion of mitochondrial outer membrane and inner membrane, respectively [41,42]. Loss of function of either of these proteins leads to neurodegenerative diseases, CMT2A and optic atrophy, respectively [43,44]. The loss-of-function mutants of Mfn2 or Opa1 reduce mitochondrial fusion and also cause increase in ROS levels [37,38]. We find that depletion of mitofusin increases superoxide. Can the increase in superoxide lead to increase in cyclin B? How may this influence the G_2_/M checkpoint?

Superoxide is known to be a mitogenic species [45]. Superoxide is quenched by the activity of two superoxide dismutase enzymes, SOD1 and MnSOD. It has been hypothesized that increased content of superoxide, and the related species hydrogen peroxide, could induce proliferation through various signaling mechanisms [46,47]. This is supported by the observation that the loss of MnSOD (implying increased superoxide) converts quiescent cells into proliferative cells by upregulating cyclin B [48]. Conversely, the overexpression of MnSOD (implying greater scavenging and reduced content of superoxide) downregulates cyclin B [48] and inhibits cell growth [49]. Superoxide may impinge on cyclin B through multiple pathways including cyclic nucleotides [47], PTEN oxidation and activation of Akt by phosphorylation [50], also involving the transcriptional activity of NF-Y [51]. The detailed mechanism of this however needs to be investigated.

During a normal cell cycle, where mitochondrial fusogens are not manipulated, at the G_2_/M transition cyclin B translocates to mitochondria [52]. Mitochondrial cyclin B increases mitochondrial superoxide output in two ways. First, by activating respiration [52] and second, by causing degradation of mitofusin through phosphorylation thus inhibiting mitochondrial fusion [7,37,38]. This critical level (concentration or content) of mitochondrial superoxide, which is specifically achieved during G_2_/M could then exert a transcriptional activity supporting production of more cyclin B and hence progression of cells through mitosis. Cyclin B and mitochondrial superoxide could form a self-amplifying loop sustaining mitotic progression. In our experiments, when mitofusin is depleted, we speculate that the content of superoxide radicals may cross this critical level, even though the cells are not in G_2_/M. The resultant constitutive expression of cyclin B would then lead to aberrant cell cycle progression, i.e, accelerated progression through S-phase. Usually, the activity of the anaphase-promoting complex / cyclosome (APC/C) reduces cyclin B levels thereby triggering anaphase. The nature of the cyclin B-mitochondrial superoxide loop indicates that the normal degradation of cyclin B could be probably delayed in mitofusin depletions. This is consistent with the delayed mitotic exit we observe. It is interesting to note that the inactivation of mitofusin is associated with lung cancers [53], breast cancer [54], colorectal cancer [55] and hepatocellular carcinoma [56]. While Ras and Raf are implicated in these, especially where Mfn2 is concerned, our results indicate there could be another pathway involving mitochondrial superoxide by which cancer cells could up-regulate cyclin B and hyperproliferate. These considerations suggest that up-regulating mitochondrial fusion could be a viable cancer therapy.

The control of cell cycle by mitochondrial fragmentation has certain similarities, but significant differences compared to the Golgi mitotic checkpoint. Golgi ribbon severing is brought about by the activity of BARS [11] and GRASP65 [12], and blocking the activity of BARS or GRASP 55/65 leads to reduced recruitment and impaired activation of Aurora-A at the centrosome [13], which prevents activation of cyclin B-Cdk1 and hence functions as a checkpoint. The similarities between mitochondria and Golgi are the change in mitochondria from tubular morphology to fragmented morphology at the G_2_/M transition, and the correlation of cyclin B content with fragmented morphology. The major differences are that mitochondrial fragmentation does not constitute a checkpoint (at least in *Drosophila*), and the observation that a product of mitochondrial function, viz. superoxide may be involved in mediating the effect on the cell cycle. This interpretation is strengthened by our experiments involving Drp1 overexpression. Overexpressing Drp1 increases mitochondrial fragmentation, but does not reduce mitochondrial fusion. Hence, the steady-state levels of superoxide would be maintained near normal levels. In this scenario, though the mitochondria are fragmented, the cell cycle would progress normally. This agrees with the model described above in which increased superoxide content could up-regulate cyclin B.

The fragmentation of reticular membrane-bound organelles and compartments is likelier to lead to more equitable inheritance of the contents into daughter cells. This line of thought is supported by a recent study with Myo19 [57]. Myo19, a myosin localizing to mitochondria, mediates equitable distribution of the organelle between daughter cells; and depletion of Myo19 results in a failure in cell division. This block in distribution of the organelles can be overcome by inducing mitochondrial fragmentation. It will be interesting to test if mitotic fragmentation of the endoplasmic reticulum also constitutes a G_2_/M checkpoint like the Golgi, or affects the cell cycle progression through its function.

## Supporting Information

S1 Fig Regulation of cell cycle progression in *Drosophila melanogaster*. Cell cycle in *Drosophila* is regulated at the G_1_-S (A) and G_2_/M (B) transitions. (A) Cyclin E expression in the late G_1_ phosphorylates the retinoblastoma protein RBF1, releasing E2F1. E2F1 forms a complex with dDP that supports transcription and further expression of cyclin E. Dacapo is a negative regulator of the cyclin E-Cdk2 complex, and hence of the G_1_-S transition. (B) Cells in G_2_ are prevented from entering mitosis by the activity of the kinases Wee1 and Myt1. Wee1 is cytosolic while Myt1 is membrane associated. Among other targets, they phosphorylate and inactivate Cdk1. Independently, dE2F-dDP upregulate the Cdc25-phosphatase, String. String removes the inhibitory phosphate bound to the ATP-binding site of Cdk1, thereby activating it. Simultaneously, Fos upregulates cyclin B. Formation of the active cyclin B-Cdk1 complex drives the cell into mitosis.
(TIF)

S1 Table *Drosophila* stocks used in this work. (DOCX)

S2 Table Antibodies used in this work. (DOCX)

S3 Table Chemicals—origin and use. (DOCX)
