# Supplementary figures and images for: Mitochondrial Fragmentation Due to Inhibition of Fusion Increases Cyclin B through Mitochondrial Superoxide Radicals
Source: PLoS One. 2015 May 22;10(5):e0126829. doi: 10.1371/journal.pone.0126829 (PMC4441460; doi:10.1371/journal.pone.0126829)

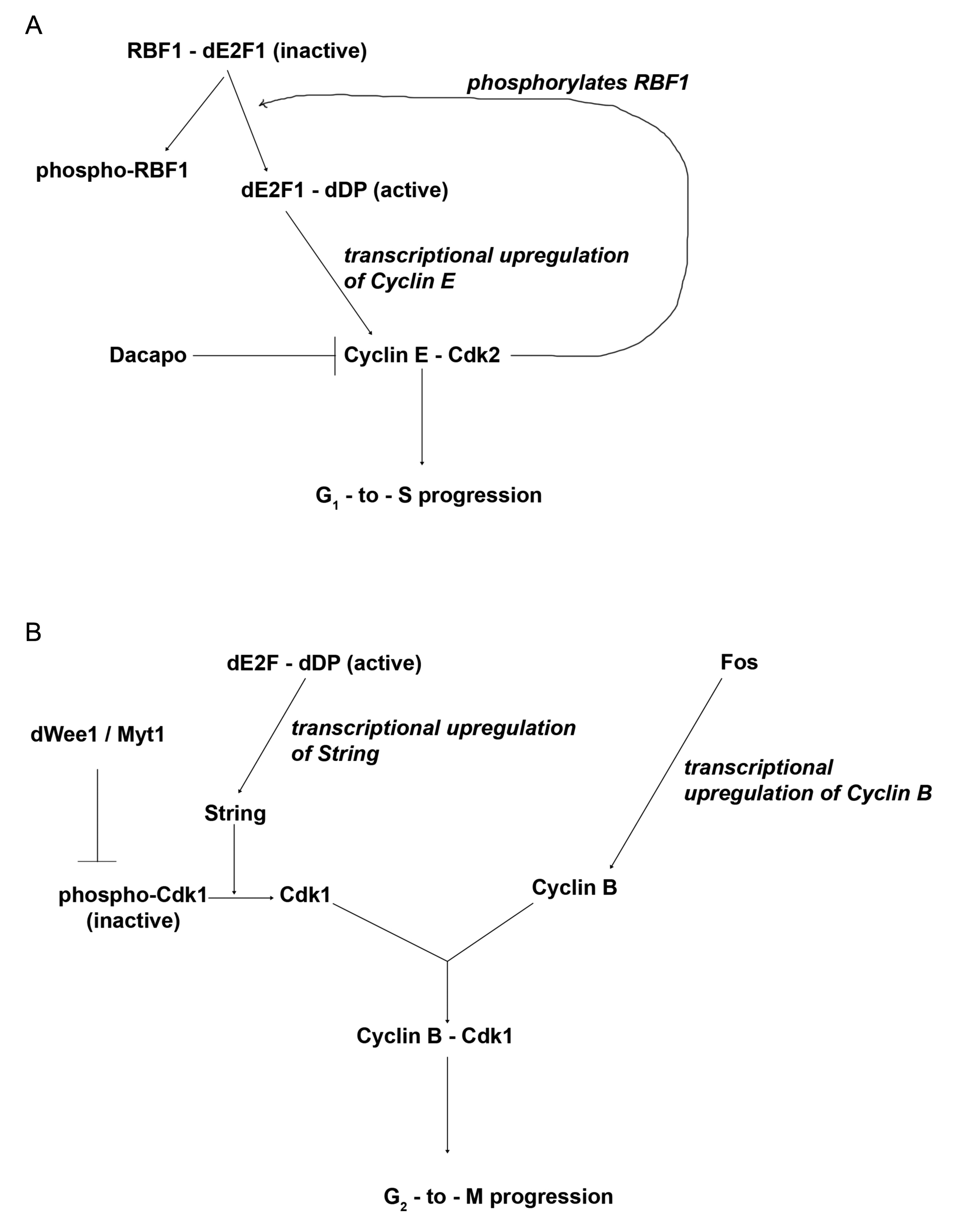

Supplement: S1 Fig — Cell cycle in Drosophila is regulated at the G1-S (A) and G2/M (B) transitions. (A) Cyclin E expression in the late G1 phosphorylates the retinoblastoma protein RBF1, releasing E2F1. E2F1 forms a complex with dDP that supports transcription and further expression of cyclin E. Dacapo is a negative regulator of the cyclin E-Cdk2 complex, and hence of the G1-S transition. (B) Cells in G2 are prevented from entering mitosis by the activity of the kinases Wee1 and Myt1. Wee1 is cytosolic while Myt1 is membrane associated. Among other targets, they phosphorylate and inactivate Cdk1. Independently, dE2F-dDP upregulate the Cdc25-phosphatase, String. String removes the inhibitory phosphate bound to the ATP-binding site of Cdk1, thereby activating it. Simultaneously, Fos upregulates cyclin B. Formation of the active cyclin B-Cdk1 complex drives the cell into mitosis. (TIF) [file pone.0126829.s001.tif]
