# Supplementary material for: Mitochondrial Fragmentation Due to Inhibition of Fusion Increases Cyclin B through Mitochondrial Superoxide Radicals
Source: PLoS One. 2015 May 22;10(5):e0126829. doi: 10.1371/journal.pone.0126829 (PMC4441460; doi:10.1371/journal.pone.0126829)
Supplement: S1 Table — (DOCX) [file pone.0126829.s002.docx]

**S1 Table. *Drosophila* stocks used in this work**

| Genotype | Chromosome number | Source  (Stock number) | Comments | Reference |
| --- | --- | --- | --- | --- |
| Canton-S Benzer  (Cs-Bz) |  |  | Wild type strain |  |
| UAS-mito::GFP /CyO | II | BDSC 8442 | Overexpression construct for n-terminal 31 amino acids of human cytochrome c oxidase subunit VII followed by a gene encoding GFP (S65T) | Pilling et al., 2006 |
| UAS-H2B::mRFP | III | Courtesy Maithreyi Narasimha | Overexpression construct for histone H2B tagged with monomeric RFP |  |
| UAS-myr::RFP | II | BDSC 7118 | Overexpression construct for monomeric RFP targeted to the membrane by myristoylation | Speder et al., 2006 |
| UAS-cat | II | BDSC 24621 | Overexpression construct for catalase | Owusu-Ansah and Banerjee, 2009 |
| UAS-cycB | II | BDSC 6626 | Overexpression construct for *Drosophila* cyclin B | Khurana et al., 2006 |
| UAS-stg | II | BDSC 4777 | Overexpression construct for *Drosophila* String | Mata et al., 2000 |
| UAS-dap |  | NCBS UR10-09 | Overexpression construct for *Drosophila* Dacapo | Lane et al., 1996 |
| UAS-Drp1::GFP | II | Courtesy S. Vivek Raj | Overexpression construct for *Drosophila* Drp1 tagged with EGFP |  |
| UAS-marf IR | II | VDRC 105261/KK | Construct encoding dsRNA targeting *marf* |  |
| UAS-mitoPLD IR | III | VDRC 48764/GD | Construct encoding dsRNA targeting *mitoPLD* | Muliyil et al., 2011 |
| UAS-myt1 IR | II | VDRC 105157/KK | Construct encoding dsRNA targeting *myt1* |  |
| UAS-OR47 IR | III | VDRC 9354/GD | Construct encoding dsRNA targeting *OR47b* |  |
| Cg-GAL4 | II | BDSC 7011 | GAL4 expresses in hemocytes | Asha et al., 2003 |
| Cg-GAL4,  UAS-mito::GFP /CyO | II | Recombined by Ruchika Anand | GAL4 causes expression of mitoGFP in hemocytes |  |
| sd-GAL4 | X | BDSC 8609 | GAL4 expressed in wing pouch and lateral margins of wing disc | Roy et al., 1997 |
| H2B::GFP |  | Courtesy G V Shivashankar | Construct for constitutive expression of histone H2B tagged with GFP(S65T) |  |

**Supporting References**

**Pilling, A. D., Horiuchi, D., Lively, C. M., and Saxton, W. M.** (2006). Kinesin-1 and Dynein are the primary motors for fast transport of mitochondria in *Drosophila* motor axons. *Mol. Biol. Cell*. **17**, 2057-2068.

**Speder, P., Adam, G., and Noselli, S.** (2006). Type ID unconventional myosin controls left-right asymmetry in *Drosophila*. *Nature*. **440**, 803-807.

**Owusu-Ansah, E., and Banerjee, U.** (2009). Reactive oxygen species prime *Drosophila* haematopoietic progenitors for differentiation. *Nature*. **461**, 537-541.

**Khurana, V., Lu, Y., Steinhilb, M. L., Oldham, S., Shulman, J. M., and Feany, M. B.** (2006). TOR-mediated cell-cycle activation causes neurodegeneration in a *Drosophila* tauopathy model. *Curr. Biol*. **16**, 230-241.

**Mata, J., Curado, S., Ephrussi, A., and Rorth, P.** (2000). Tribbles coordinates mitosis and morphogenesis in *Drosophila* by regulating string/CDC25 proteolysis. *Cell*. **101**, 511-522.

**Lane, M. E., Sauer, K., Wallace, K., Jan, Y. N., Lehner, C. F., and Vaessin, H.** (1996). Dacapo, a cyclin-dependent kinase inhibitor, stops cell proliferation during *Drosophila* development. *Cell.* **87**, 1225-1235.

**Muliyil, S., Krishnakumar, P., and Narasimha, M.** (2011). Spatial, temporal and molecular hierarchies in the link between death, delamination and dorsal closure. *Development.* 138, 3043-3054.

**Asha, H., Nagy, I., Kovacs, G., Stetson, D., Ando, I., and Dearolf, C. R.** (2003). Analysis of Ras-induced overproliferation in *Drosophila* hemocytes. *Genetics*. **163**, 203-215.

**Roy, S., Shashidhara, L. S., and VijayRaghavan, K.** (1997). Muscles in the *Drosophila* second thoracic segment are patterned independently of autonomous homeotic gene function. *Curr. Biol.* **7**, 222-227.
