# Supplementary material for: Mitochondrial Fragmentation Due to Inhibition of Fusion Increases Cyclin B through Mitochondrial Superoxide Radicals
Source: PLoS One. 2015 May 22;10(5):e0126829. doi: 10.1371/journal.pone.0126829 (PMC4441460; doi:10.1371/journal.pone.0126829)
Supplement: S2 Table — (DOCX) [file pone.0126829.s003.docx]

**S2 Table. Antibodies used in this work**

| **Antibody (α-antigen)** | **Source** | **Application** | **Dilution, time, temperature** | **Fixation, Permeabilization** |
| --- | --- | --- | --- | --- |
| α-phospho (Ser10)  Histone H3 | Millipore  #06-570 | Immunofluorescence microscopy of  hemocytes | 1:500,  4^O^C,  overnight. | 100% acetone, 25^O^C, 6 min |
| α-phospho (Ser10)  Histone H3 | Millipore  #06-570 | Immunofluorescence microscopy of wing disc | 1:500,  4^O^C,  overnight. | Fix: 4% paraformaldehyde,  0.3% TritonX-100 in 1x D-PBS, 25^O^C, 30 min.  Permeabilize: 0.3% TritonX-100 in 1x D-PBS, 25^O^C, 30 min. |
| α-phospho (Ser10)  Histone H3 | Millipore  #06-570 | Flow cytometry of S2R^+^ cells | 1:100,  25^O^C,  30 min. | Fix: 95% chilled ethanol in 1x HBSS, on ice, 30 min.  Permeabilize: 0.25% TritonX-100, 1% bovine serum albumin in 1x HBSS, on ice, 15 min. |
|  |  |  |  |  |
| α-Cyclin B | DSHB  #F2F4 | Flow cytometry of S2R^+^ cells | 1:10  25^O^C,  30 min. | Fix: chilled 1% paraformaldehyde in 1x HBSS, on ice, 30 min.  Permeabilize: 0.1% Tween-20, 1% bovine serum albumin in 1x HBSS, on ice, 30 min. |
|  |  |  |  |  |
| α-BrdU | DSHB  #G3G4 | Flow cytometry of S2R^+^ cells | 1:10  25^O^C,  30 min  in the dark | Fix: 95% chilled ethanol in 1x HBSS, on ice, 60 min.  Rehydrate: 1x HBSS, 25^O^C,  5 min  Permeabilize: 2N HCl, 0.1% Tween-20, 1% bovine serum albumin in 1x HBSS, 25^O^C, 30 min. |
