# Supplementary material for: Mitochondrial Fragmentation Due to Inhibition of Fusion Increases Cyclin B through Mitochondrial Superoxide Radicals
Source: PLoS One. 2015 May 22;10(5):e0126829. doi: 10.1371/journal.pone.0126829 (PMC4441460; doi:10.1371/journal.pone.0126829)
Supplement: S3 Table — (DOCX) [file pone.0126829.s004.docx]

**S3 Table. Chemicals – origin and use.**

| **Reagent** | **Source** | **Stock solution** | **S2R+** |
| --- | --- | --- | --- |
| MitoTracker DeepRed | Life technologies #M22426, | 1 mM in DMSO | 20 nM, 15 min  in growth medium – S2R^+^  2 nM, 15 min  in SCM – hemocytes |
| Hoechst 33343 | Sigma  # B2261 | 1 mg/ml in pure water | 5 μg/ml, 15 min  in growth medium – S2R^+^  5 μg/ml, 15 min in SCM – hemocytes |
| Benzylpenicillin | Alembic Ltd |  |  |
| Streptomycin | Sarabhai Piramal pharmaceuticals Pvt. Ltd. |  |  |
| L-glutamine | Sigma #G3126 |  |  |
| 5’-bromo-2’-deoxyuridine  (BrdU) | Sigma  #B9285 | 10 mM in  1x HBSS buffer | Use at 60 μM, 1 h in growth medium – S2R^+^ |
| Dihydroethidium | Fluka #37291 | 10 mM in DMSO | 2 μM, 15 min  in SCM - hemocytes |
| Paraformaldehyde | Sigma #P6148 | 20% in buffer pH 6.9 | 2.5%, 15 min in  1x M1 buffer – both cell types |
| Propidium iodide | Sigma #P4170 | 0.5 mg/mL in  5 mM trisodium citrate dihydrate |  |
| RNase | Sigma # R4875 | 1 mg/mL in 100 mM NaCl, 2 mM MgCl_2_, 0.25 mM EDTA, 10 mM PIPES |  |
| Triton-X-100 | Sigma #X100 |  |  |
| Tween-20 | Sigma  #P5927 |  |  |
| Hank’s balanced salt solution HBSS | Life technologies 14185-045 |  |  |

100 x penicillin-streptomycin-glutamine solution

10^4^ units/ml of benzylpenicillin, 10^4^ μg/ml of streptomycin, 30 mg/mL of L-glutamine in pure water.

10X M1

NaCl (Fisher Scientific) 87.66 g, KCl (Fisher Scientific) 3.727 g, MgCl_2_.6H_2_O (Fisher Scientific) 2.03 g, CaCl_2_.2H_2_O (Sigma Aldrich) 1.4702 g, HEPES (Sigma Aldrich) 47.60 g.

Milli-Q water to bring volume to 1000 ml. Dilute to 1x, adjust pH to 6.9 using 10N NaoH.
